# MUC4 and MUC5AC are highly specific tumour-associated mucins in biliary tract cancer

**DOI:** 10.1038/sj.bjc.6604364

**Published:** 2008-05-13

**Authors:** W R Matull, F Andreola, A Loh, Z Adiguzel, M Deheragoda, U Qureshi, S K Batra, D M Swallow, S P Pereira

**Affiliations:** 1 The Institute of Hepatology, Division of Medicine, Royal Free & University College London Medical School, London, UK; 2 Galton Laboratory, Department of Biology, University College London, London, UK; 3 Department of Histopathology, University College London Hospitals NHS Foundation Trust, London, UK; 4 Oncology Department, Cancer Research UK Targeting and Imaging Group, Royal Free University College London Medical School, London, UK; 5 Eppley Cancer Institute, Department of Biochemistry and Molecular Biology, University of Nebraska, Omaha, NE, USA; 6 Department of Gastroenterology, University College London Hospitals NHS Foundation Trust, London, UK

**Keywords:** biliary tract cancer, mucin glycoprotein, tumour marker, cholangiocarcinoma

## Abstract

Alterations in epithelial mucin expression are associated with carcinogenesis, but there are few data in biliary tract cancer (BTC). In pancreatic malignancy, MUC4 is a diagnostic and prognostic tumour marker, whereas MUC5AC has been proposed as a sensitive serological marker for BTC. We assessed MUC4 and MUC5AC expression in (i) prospectively collected bile and serum specimens from 72 patients with biliary obstruction (39 BTC) by real-time reverse transcriptase–PCR (qPCR) and western blot analysis, and (ii) 79 archived biliary tissues (69 BTC) by immunohistochemistry. In bile, MUC4 protein was detected in 27% of BTC and 29% of primary sclerosing cholangitis (PSC) cases, but not in other benign and malignant biliary diseases (*P*<0.01 and *P*=0.06). qPCR revealed a 1.9-fold increased MUC4 mRNA expression in BTC patients’ bile compared with benign disease. In archived tissues, MUC4 protein was detected in 37% of BTC but in none of the benign samples (*P*=0.03). In serum, MUC5AC was found exclusively in BTC and PSC sera (44% and 13%, respectively; *P*<0.001 for BTC *vs* non-BTC) and correlated negatively with BTC survival. Biliary MUC4 and serum MUC5AC are highly specific tumour-associated mucins that may be useful in the diagnosis and formulation of therapeutic strategies in BTC.

Biliary tract cancer (BTC; gallbladder or cholangiocarcinoma) has a poor prognosis. Surgery is associated with 5-year survival rates of 24–40% (Seyama and Makuuchi, 2007), but more than 80% of patients are considered unresectable at the time of diagnosis (Jarnagin *et al*, 2001) and have a median survival of approximately 6–9 months (Khan *et al*, 2002).

The early detection of BTC remains a clinical challenge. Even in advanced disease, tissue diagnosis is often difficult and the sensitivity is low (Khan *et al*, 2002; Malhi and Gores, 2006). The most commonly used BTC tumour markers, carbohydrate antigen (CA)19-9 and carcinoembryonic antigen, are of limited use in patients with indeterminate biliary strictures (Nehls *et al*, 2004) and there is a need for better diagnostic tests.

Alterations in the epithelial mucin glycoprotein profile are associated with malignant disease. In pancreatic tissue, of the same embryological origin as biliary epithelium, MUC4 expression is absent in benign disease, but is present in 90% of adenocarcinomas and is associated with reduced survival (Moniaux *et al*, 2004a; Saitou *et al*, 2005). In BTC, recent studies from east Asia suggest that MUC4, as well as MUC5AC, may be suitable candidate tumour markers (Shibahara *et al*, 2004; Tamada *et al*, 2006). In northern Thailand, a region with a high incidence of BTC, MUC5AC protein was detected in the sera of 63% of 179 patients with BTC, but in none of 74 healthy controls (Wongkham *et al*, 2003).

To date, there have been no studies of serum MUC5AC in other BTC patient populations and no prospective studies of mucin expression in bile. To test the hypothesis that MUC4 and MUC5AC are tumour-associated mucins in BTC, we characterised expression of MUC4, MUC5AC and MUC5B (present in normal biliary epithelium) by immunohistochemistry in archival biliary tissue and by western blotting and real-time reverse transcriptase–polymerase chain reaction (rt RT–PCR) in bile and serum samples.

## MATERIALS AND METHODS

### Patients

Over a 12-month period, bile and serum samples were collected prospectively from 72 adult patients undergoing endoscopic retrograde cholangiopancreatography (ERCP) or percutaneous transhepatic cholangiography for evaluation of indeterminate or established benign and malignant biliary tract strictures. A diagnosis of malignancy was made by positive cytology and/or biopsy, or evidence of disease progression on imaging. Benign disease was established by negative pathology and a minimum of 12-month clinical follow-up.

Bile samples (5 ml) were aspirated proximal to the stricture via a standard ERCP catheter, transferred into cryovials and placed immediately in liquid nitrogen. All clinical specimens were stored at −70°C.

The study was approved by the Joint UCLH/UCL Committee on the Ethics of Human Research (reference no. 04/0028), and all patients gave written informed consent.

### Mucin glycoprotein concentrations in bile and serum

Agarose (2%) gel electrophoresis (with 0.1% sodium dodecyl sulphate (SDS)) was performed using 10 mM 1,4-dithiothreitol (DTT) (all from Sigma-Aldrich, Steinheim, Germany). Freshly thawed bile samples were diluted 1 : 2 in 1% SDS dissolving buffer (40 mM Tris-acetate, 1 mM EDTA, 30% glycerol, 0.002% bromophenol), and a final volume of 30 *μ*l was loaded per well. Bile protein concentrations were measured using the bicinchoninic acid protein assay (Pierce, Rockford, IL, USA). Serum samples were diluted 1 : 2 in a 2% SDS dissolving buffer (as above) and 20 *μ*l loaded per well. After electrophoresis, polyvinylidene difluoride membrane (Hybond™-P; Amersham Pharmacia Biotech, Little Chalfont, UK) vacuum blotting (in 0.6 M sodium chloride/60 mM sodium citrate transfer buffer; suction pressure of 40 mbar) was performed. We used the following primary antibodies: two monoclonal MUC4 mouse antibodies, clone 8G7 (Moniaux *et al*, 2004b), detecting a 16-amino-acid epitope in the tandem repeat ectodomain (Batra's Lab; concentration 1 : 1000), and clone 1G8 (Zhang *et al*, 2005), directed towards an epitope from the membrane-associated subunit (Zymed®-affinity purified; Invitrogen, Paisley, UK; also supplied by K Carraway, Miami, FL, USA; concentration 1 : 400), a single polyclonal MUC5AC rabbit antiserum, Lum5-1 EU-batch (Hovenberg *et al*, 1996) (supplied by I Carlstedt, Lund, Sweden; concentration 1 : 600), and a single monoclonal mouse MUC5B antibody, clone EU-MUC5Ba (Rousseau *et al*, 2003) (concentration 1 : 200), both detecting peptide sequences in non-glycosylated domains in the main tandem repeat regions.

**Figure 1:**
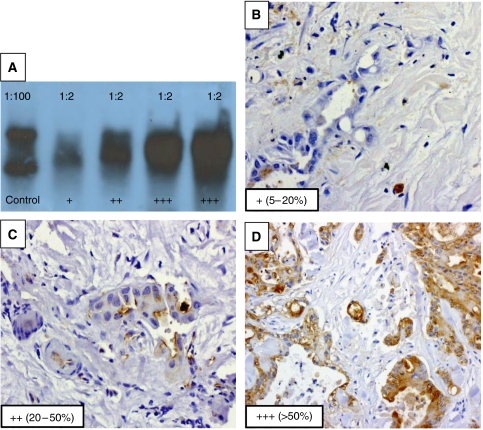
(**A**) Semiquantitative western blot assessment score – strength of band staining in relation to control sample – example with the monoclonal 8G7 clone MUC4 antibody (1 : 1000 dilution) on breast milk control (1 : 100 dilution) and four different bile samples (1 : 2 dilutions) (on 2% agarose (0.1% SDS) gel and 10 mM DTT). (**B**–**D**) Semiquantitative immunohistochemistry assessment score – percentage of positively stained malignant cells – example with the monoclonal 1G8 clone MUC4 antibody (1 : 250 dilution) on three different biliary tissue specimens (at × 400 magnification).

Four-hour first antibody incubation was followed by phosphate-buffered saline (PBS) washes (plus 0.05% Tween® 20), 1-h incubation with the appropriate secondary antibodies (rabbit anti-mouse or swine anti-rabbit (Dako A/S, Glostrup, Denmark)), further PBS/Tween washes and final chemoluminescence detection (ECL™-Western-blotting-detection reagents; Amersham Biosciences, Little Chalfont, UK). The strength of the bands was assessed in relation to the positive control samples (breast milk (1 : 100 dilution) for MUC4, sputum (1 : 10) for MUC5AC, saliva (1 : 200) for MUC5B), using a semiquantitative assessment score (negative, +, ++ and +++), as shown in Figure 1. Band signals of one + strength or greater were considered positive in the analysis.

### Mucin mRNA expression (by rt RT–PCR)

#### RNA extraction

Total RNA was extracted from 1 ml aliquots of thawed bile using TRI reagent (Ambion Inc., Austin, TX, USA), according to the manufacturer's protocol (two volumes of TRI reagents per one volume of bile). To remove contaminating DNA, RNA samples were further (a) DNase-I-digested using Turbo-DNase-free kit (Ambion Inc.) and (b) purified using RNeasy-Mini-Elute kit (Qiagen Ltd, Crawley, UK).

#### cDNA synthesis

Purified total RNA samples were reverse-transcribed using iScript™-Select-cDNA-Synthesis kit (Bio-Rad Laboratories, Hemel Hempstead, UK), according to the manufacturer's protocol. Reactions were primed with random hexamers; the total volume per reaction was 20 *μ*l.

#### Real-time PCR amplification

PCR was performed in a total volume of 25 *μ*l using qPCR-Master-Mix-plus-dNTP kit (Eurogenetec, Southampton, UK) and analysed on an Applied Biosystems 7500 Real-Time PCR system (Applied Biosystems, Foster City, CA, USA). A 1 *μ*l portion of cDNA per sample was used as template. All amplifications were performed in duplicate. The thermal cycling conditions included 50°C for 2 min and 95°C for 10 min, followed by 40 cycles of 95°C for 15 s and 60°C for 1 min.

#### Primers and probes

Primers and probe sets for MUC4 and MUC5AC were sourced from published reports (Argüeso *et al*, 2002) and synthesised by Eurogenetec:

MUC4 (GenBank™ accession number AF058803): forward primer 5′-1499GCCCAAGCTACAGTGTGACTCA1520-3′; reverse primer 5′-1600ATGGTGCCGTTGTAATTTGTTGT1578-3′; probe 5′(Yakima Yellow)-1532CGGCCACATCCCCATCTTCTTCAC1555(BHQ-1)-3′; located in the C-terminal section (exon 25) of MUC4.

MUC5AC (GenBank accession number Z48314): forward primer 5′-273TCCACCATATACCGCCACAGA293-3′; reverse primer 5′-375TGGACGGACAGTCACTGTCAAC354-3′; probe 5′(Dragon Fly)-297CTCGCTGGCCATTGCTATTATGCCC321(BHQ-2)-3′; located in the first exon after the tandem repeat domain of MUC5AC.

The endogenous human glyceraldehyde-3-phosphate dehydrogenase (GAPDH) control reagents were purchased from Applied Biosystems.

#### Real-time RT–PCR

Quantitative values for mucin expression were obtained by TaqMan® methodology from identification of the cycling point when PCR product is detectable (threshold cycle or *C*_*T*_ value). To normalise the amount of total RNA present in each reaction, we amplified the housekeeping gene GAPDH. Results are expressed as levels of MUC(s) mRNA, relative to normal bile samples (the calibrator), chosen to represent 1 × expression of this gene. The pathological bile samples express *n*-fold MUC(s) mRNA relative to the calibrator. The amount of target, normalised to an endogenous reference (GAPDH) and relative to the calibrator, is defined by the double-delta *C*_*T*_ (ΔΔ*C*_*T*_) method as described by Livak and Schmittgen (2001). To ascertain the origin of RNA in bile, we tested samples for expression of CK-19 and CD45, markers for biliary epithelium and leukocytes, respectively.

### Mucin immunohistochemistry

Archival formalin-fixed biliary tissue from 69 patients with BTC and 10 without biliary disease were identified from the University College Hospital tissue bank. The following monoclonal antibodies were employed: 1G8 clone against MUC4 (see above), 21M1 clone against MUC5AC (Nollet *et al*, 2002) (supplied by J Bara, Paris, France, which detects the vWF-A3uD4 domain) and EU-MUC5Bb clone against MUC5B (see above). The following normal tissues were used as positive antibody controls: stomach for MUC5AC, bronchus for MUC4 and MUC5B.

Paraffin sections of 3 *μ*m were dewaxed with Histoclear™ (RA Lamb, Eastbourne, UK) and dehydrated in graded alcohols. Endogenous peroxidase was blocked with 1.5% hydrogen peroxide diluted in methanol for 10 min, followed by a reduction step in 10 mM DTT (Sigma-Aldrich) in 0.1 M Tris/HCl buffer, pH 8, at 25°C for 30 min and treatment with 0.025 M of the alkylating agent iodoacetamide (Sigma-Aldrich) in 0.1 M Tris/HCl, pH 8.0, for another 30 min. No reduction was required for MUC4. Sections were then washed with Tris-buffered saline (TBS). For improved antigen retrieval for MUC4 and MUC5B stainings, tissues were microwaved in 0.01 M citrate buffer, pH 6, on high power for 10 min. For MU5AC staining, tissue was subjected to trypsinisation for 5 min at 37°C with 50 mg chymotrypsin (Sigma-Aldrich) dissolved in 1 mM calcium chloride, pH 7.8. Liver tissues were blocked for endogenous biotin using Dako's biotin-blocking kit (Dako A/S). Vectastain Elite-ABC-mouse-IgG kit (Vector Laboratories, Burlingame, CA, USA) was used for immunohistochemical staining. Sections were washed again in TBS and incubated in normal horse serum for 20 min. This was followed by primary antibody exposure: 1G8 (MUC4) diluted at 1 : 250 (purified ascites; 0.5 mg ml^−1^); 21M1 (MUC5AC) diluted at 1 : 1 000 000 (ascites fluid); and EU-MUC5Bb (MUC5B) diluted at 1 : 100 (culture supernatant). All antibodies were incubated for 1 h at 25°C. Sections were then washed in TBS, and a horse anti-mouse biotinylated secondary antibody was applied for 30 min, followed by 30-min incubation in horseradish peroxidase-conjugated avidin–biotin complex. Sections were developed in diaminobenzidine (Dako A/S) and counterstained in haematoxylin.

All slides were assessed by an experienced pancreaticobiliary histopathologist (MD), applying a semiquantitative scoring system (negative=less than 5% of neoplastic cells stained positive, +=5–20%, ++=20–50%, +++=more than 50%), as used in previous publications (Tamada *et al*, 2006) and shown in Figure 1. High-expression samples (⩾20%) were considered positive in the analysis.

### Statistical analysis

Statistical analysis was performed using SPSS software, version 14.0 (SPSS Inc., Chicago, IL, USA). Numerical data were presented as medians with ranges or as mean values with standard errors of the mean. Intergroup comparisons were performed with Student's *t*-test for continuous variables and *χ*^2^ test for binary variables. Survival was determined from the time of diagnosis to the time of census on 30 June 2007. The Kaplan–Meier method was used for survival rate estimates. Statistical significance between groups was determined by log-rank or generalised Wilcoxon test. A *P*-value of less than 0.05 was considered significant.

## RESULTS

### Clinical characteristics

Of the 72 patients from whom bile and blood samples were collected prospectively, 39 patients (54%) were diagnosed with BTC, 8 (11%) had non-BTC malignant conditions, 7 (10%) had primary sclerosing cholangitis (PSC) and 18 (25%) had other benign conditions.

Demographic, biochemical and clinical data of the patients, as well as location and clinical T-staging (American Joint Committee on Cancer (AJCC), 2002) of the BTC group, are summarised in Table 1.

Complete survival data were available for all 39 BTC patients. The overall median survival was 11.4 months (range: 0.3–46.3 months; 95% CI: 6.4–16.5 months) from the time of BTC diagnosis.

### Biliary MUC4 protein expression is highly BTC specific

#### Immunohistochemistry

Archival tissue was obtained from 69 BTC patients (36 men and 33 women, median 69 years (range: 45–85 years), 46 perihilar CCA, 15 gallbladder carcinoma, 5 distal extrahepatic and 3 intrahepatic CCA) and from 10 patients without biliary disease (7 women and 3 men, median age 67 years (range: 45–88 years), normal gallbladder and bile duct specimens from pancreaticoduodenectomies). Fifty-seven (83%) of the malignant specimens were from patients with advanced cancer stage (⩾T3=organ and/or large vascular structure invasion). Twelve (17%) had known distant metastases at the time. The cancers were graded as well differentiated in 12% (8 out of 69), moderately differentiated in 71% (49 out of 69) and poorly differentiated in 17% (12 out of 69).

**Figure 2:**
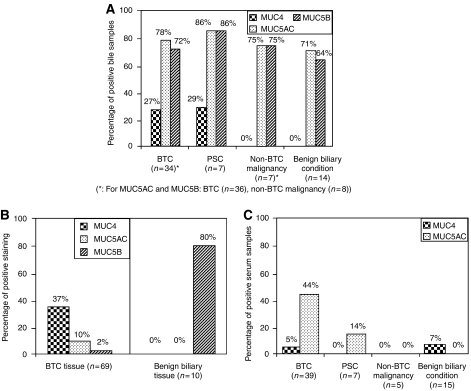
Mucin protein expressions in bile, biliary tissue and serum of patients with biliary strictures (evaluated by presence and absence of staining). (**A**) Western blotting of MUC4, MUC5AC and MUC5B expression in bile samples of BTC and non-BTC patients. MUC4 expression was more frequently seen in bile of BTC patients (*P*<0.01). (**B**) Immunohistochemistry of MUC4, MUC5AC and MUC5B expression in BTC and benign biliary tissues. Again, MUC4 expression was more frequently seen in BTC tissues (*P*=0.03). (**C**) Western blotting of MUC4 and MUC5AC expression in serum samples of BTC and non-BTC patients. In serum, MUC5AC expression was statistically significantly more frequently seen in BTC patients (*P*<0.01), but no such association was seen for MUC4 in serum.

In BTC specimens, 37% (26 out of 69) showed MUC4 protein expression, 10% (7 out of 69) MUC5AC and 2% (1 out of 69) MUC5B. In benign biliary tissue, 80% (8 out of 10) showed MUC5B expression, but neither MUC4 nor MUC5AC expression was seen (Figure 2). Thus, MUC4 protein expression was associated with BTC (*P*=0.03), whereas MUC5B protein was more commonly expressed in benign biliary disease (*P*<0.001). There was no correlation between the strength of mucin staining and clinical cancer stage for either MUC4 or MUC5AC.

Survival data were available for 94% (65 out of 69) of the retrospective BTC cohort. The overall median survival was 9 months (range: 0.2–46.3 months; 95% CI: 4.5–13.4 months). There was no survival difference between BTC patients with MUC4-positive and MUC4-negative disease (*P*=0.81). Equally, there were no survival differences seen for MUC5AC- or MUC5B-positive and negative BTC patients.

#### Western blot analysis

Sixty-five bile samples (90%) were available for western blot and mRNA expression analysis. Nine of 34 (27%) BTC and 2 of 7 (29%) PSC bile samples were positive for MUC4 protein, as compared to none of the 7 non-BTC malignancy or 14 benign biliary condition samples (3 bile samples not interpretable for MUC4 owing to nonspecific antibody detection). This difference reached statistical significance for BTC (*P*<0.01) but not for PSC (*P*=0.06). Normalising bile samples according to protein content (range: 1.3–5.2 *μ*g *μ*l^−1^) did not affect the overall outcome of the western blot analysis. There was no difference in biliary MUC5AC and MUC5B expressions between the four disease categories (Figure 2).

Median survival for BTC patients with MUC4-positive biliary western blot result was 5.2 months (range: 1.3–26.6 months; 95% CI: 4.6–5.8 months) compared with 11.8 months (range: 0.6–46.3 months; 95% CI: 7.8–15.2 months) for MUC4-negative BTC patients (*P*=0.30).

### Biliary MUC4 and MUC5AC mRNA expression is raised in PSC and biliary malignancy

**Figure 3:**
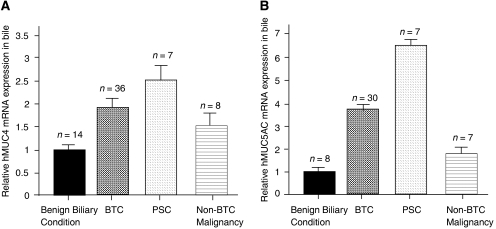
Human (h) MUC4 and MUC5AC mRNA expressions in clinical bile samples, normalised to GAPDH and relative to the calibrator ‘benign biliary conditions’ (=normal). (**A**) Relative hMUC4 mRNA expression was increased in PSC and malignant biliary strictures (*P*<0.05). (**B**) Relative hMUC5AC mRNA expression was highest in PSC, followed by BTC and non-BTC malignancies, with statistically significant differences between the disease categories (*P*<0.05).

Based on the Livak calculation (see Materials and Methods), there was a 1.9-fold increase (95% CI: 1.69–2.33) in MUC4 mRNA expression in BTC bile samples (*n*=36) as compared to benign biliary condition samples (*n*=14; 95% CI: 0.86–1.16). This mRNA signal in bile originated predominantly from biliary epithelial cells, as shown by high CK-19 rather than CD45 gene expression (Supplementary Figure). Bile samples from PSC (*n*=7) and non-BTC malignancy (*n*=8) patients showed a 2.6-fold (95% CI: 2.21–3.11) and 1.5-fold (95% CI: 1.18–1.88) increase in MUC4 mRNA level over benign biliary conditions, respectively. The relative increases of MUC4 mRNA expression levels in BTC, PSC and non-BTC malignancies as compared to benign biliary conditions were statistically significant (*P*⩽0.05 for each; Figure 3).

MUC5AC mRNA was not detected in 13 of 65 bile samples (i.e., *C*_*T*_ value >40; 6 normal, 6 BTC and 1 non-BTC malignancy sample). For the remaining 52 bile samples, there was a 3.8-fold increase (95% CI: 3.33–4.43) in MUC5AC mRNA expression level in BTC bile samples (*n*=30) as compared to benign biliary condition samples (*n*=8; 95% CI: 0.83–1.21). Bile samples from PSC (*n*=7) and non-BTC malignancy (*n*=7) patients showed a 6.4-fold (95% CI: 4.75–8.61) and 1.9-fold (95% CI: 1.68–2.15) increase, respectively, in MUC5AC mRNA level over benign biliary conditions. The differences in relative MUC5AC mRNA expression levels were statistically significant for BTC, PSC and non-BTC malignancies as compared to benign biliary conditions, as well as between the disease categories (Figure 3).

### MUC5AC protein expression in serum is BTC specific

Sixty-six serum samples (92%) were available for western blot analysis. Seventeen of 39 (44%) BTC and 1 of 7 (14%) PSC sera were positive for MUC5AC protein as compared to none of the 5 non-BTC malignancy or 15 benign biliary disease serum samples. This difference reached statistical significance for BTC (*P*<0.01) but not for PSC (*P*=0.26). Serum MUC4 protein was detected in 2 of 39 (5%) BTC patients, in 1 of 15 (7%) patients with benign biliary disease and in none of the sera of PSC and non-BTC malignancy patients. These differences were not statistically significant (Figure 2).

**Figure 4:**
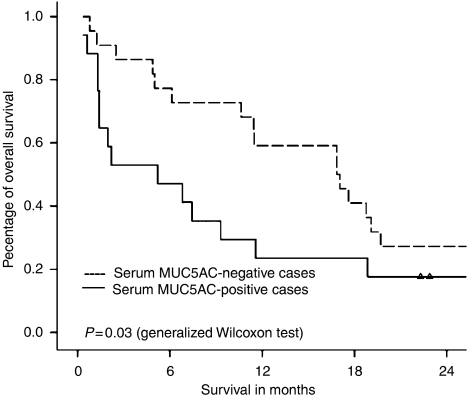
Kaplan–Meier survival curves of 39 consecutive BTC patients. Patients with MUC5AC-positive serum had a lower median survival than MUC5AC-negative patients (*P*=0.03).

The median survival (from time of BTC diagnosis) was 5.2 months (range: 0.3–31.7 months; 95% CI: 0.0–11.7 months) for patients with MUC5AC-positive sera compared with 16.9 months (range: 0.8–46.3 months; 95% CI: 9.8–23.9 months) for MUC5AC-negative BTC patients (*P*=0.03); the Kaplan–Meier survival curves are shown in Figure 4.

Combining the serum MUC5AC expression with the biliary MUC4 analysis resulted in a 14% increase in sensitivity but a 9% decrease in specificity (Table 2). The accuracy of combined testing was 69% (42 out of 61).

In patients with either biliary MUC4-positive or serum MUC5AC-positive western blot results, the median survival was 6.8 months (range: 0.3–31.7 months; 95% CI: 3.0–10.6 months) compared with 17.6 months (range: 0.8–46.3 months; 95% CI: 8.4–26.8 months) for MUC4- and MUC5AC-negative BTC patients (*P*=0.06).

## DISCUSSION

Alterations in epithelial mucin core protein and glycosylation play a role in cellular growth, differentiation, invasion and immune surveillance, and are known to influence metastases and survival for a variety of cancers (Hollingsworth and Swanson, 2004). However, there are few data, derived mainly from immunohistochemistry studies, on mucin profile changes in BTC (Lee and Liu, 2001; Shibahara *et al*, 2004).

In the present study, we detected a strong association between biliary MUC4 and serum MUC5AC protein expression and BTC. None of the specimens from patients with benign, non-PSC biliary conditions were positive for biliary MUC4 or serum MUC5AC, either by western blot analysis or by immunohistochemistry. Hence, MUC4 protein in bile and MUC5AC protein in serum are both highly specific markers of BTC. We also detected increased expressions of MUC4 and MUC5AC mRNA in BTC, although there was some discrepancy between mucin protein and mRNA analyses in bile. This may have been due to the greater sensitivity of real-time RT–PCR or perhaps the difference in probe locations, which were selected to be in regions not known to be prone to alternative splicing, a common phenomena for MUC4 (Moniaux *et al*, 2000). The two separate MUC4 antibodies used in this study detect different regions of the glycoprotein: clone 1G8 recognises an epitope from the transmembrane subunit, expected to be present in association with cellular material, whereas clone 8G7 binds to the extracellular tandem repeat domain, present in secretions. However, the epitope may be made cryptic by post-translational alterations including glycosylation, which differs between tissues and may change in disease (Carlstedt *et al*, 1985; Hollingsworth and Swanson, 2004). Although there is little tandem repeat variation in MUC5AC, interindividual variation in MUC4 tandem repeats and the unusually alkaline and detergent properties of bile may have affected the sensitivity of epitope detection. However, the lack of detection of MUC4 protein in normal bile and only low levels of MUC4 RNA suggest that MUC4 is not synthesised to any great extent in healthy biliary epithelial cells.

Membrane-associated mucins like MUC4 have become a focus of interest, as they function as intrinsic ligands and modulate the receptor tyrosine kinase ErbB2 (Carraway *et al*, 2003), an antiapoptotic pathway used by most types of common epithelial tumours (Yarden and Sliwkowski, 2001; Funes *et al*, 2006). Therapeutic blockade of ErbB2 with the monoclonal antibody trastuzumab improves the effectiveness of chemotherapy and survival in breast cancer (Vogel *et al*, 2002). In human mammary epithelial cells, MUC4 has an antiadhesive effect and appears to promote tumour growth and progression (Pino *et al*, 2006; Singh *et al*, 2007). In human pancreatic intraepithelial neoplasia, MUC4 expression increases progressively with advanced dysplasia (Swartz *et al*, 2002), and inhibition of MUC4 by antisense RNA suppresses cell growth and metastases in pancreatic cancer cells (Singh *et al*, 2004; Moniaux *et al*, 2007). In other prospective BTC serves, there was a trend towards reduced survival in patients with MUC4-positive bile (5.0 *vs* 11.5 months). A similar survival difference was described in a Japanese population undergoing surgery for extrahepatic BTC (Tamada *et al*, 2006).

We demonstrated a 96% BTC-specificity for MUC5AC protein detection in the serum of patients with biliary tract strictures. This concurs well with results from the original Thai study (Wongkham *et al*, 2003) and those from a subsequent ELISA-based study from the same group, where serum MUC5AC positivity was associated with reduced survival (Boonla *et al*, 2003; Bamrungphon *et al*, 2007). We detected a similar significant survival difference in our study population. The relatively low level of MUC5AC positivity in the archival tissue can be explained, in part, by the use of a less sensitive MUC5AC antibody (monoclonal 21M1) for the immunohistochemistry. It is likely that the loss of cell orientation in advanced neoplasia results in ‘spilling’ of the normally apically secreted MUC5AC mucin into the circulation, which was then detectable with the more sensitive polyclonal Lum5-1 antibody.

In the present study, the specificity for BTC detection of biliary MUC4 and serum MUC5AC was superior to serum CA19-9: 93% for biliary MUC4, 96% for serum MUC5AC and 65% for serum CA19-9 (a value comparable with the published literature; Nehls *et al*, 2004). This high specificity was, however, offset by comparatively low sensitivity values of 27% for biliary MUC4 and 44% for serum MUC5AC. The sensitivity for BTC detection increased to 58% by combining the biliary MUC4 and serum MUC5AC data with a minor decrease in specificity (87%).

In conclusion, we have shown that in patients with biliary obstruction, MUC4 expression in bile and MUC5AC expression in serum are highly specific markers for BTC. The role of serum MUC5AC as a non-invasive test for BTC, including in early-stage disease, warrants further study. Targeting MUC4, with its interaction with the oncoprotein ErbB2, may be a useful therapeutic strategy in BTC.

## Figures and Tables

**Table 1:** Clinical characteristics of the 72 patients with biliary tract strictures

	**BTC (*n*=39)**	**Non-BTC (*n*=33)**	***P*-value**
Age (years)^a^	67 (34–90)	59 (23–78)	<0.01^b^
Gender (female:male)	16 : 23	18 : 15	0.25^b^
Ethnicity: Caucasian	95% (37/39)	85% (28/33)	0.75^b^
Serum bilirubin (*μ*mol l^−1^)^a^	119 (5–900)	20 (5–401)	0.01^b^
Serum CA19-9 (IU l^−1^)^a^	493 (<1–80000)	52 (<1–1354)	0.03^b^
Serum CEA (ng ml^−1^)^a^	3.1 (0.6–155)	1.8 (0.8–12.3)	0.08^b^
			
*BTC topography (*n*=39)*			
Perihilar CCA (Klatskin)	30 (77%)		
Bismuth 4	18 (46%)		
Bismuth 3	11 (28%)		
Bismuth 2	1 (3%)		
Extrahepatic CCA	5 (13%)		
Gallbladder carcinoma	4 (10%)		
			
*BTC clinical T-stage* c			
T2	2 (5%)		
T3	22 (56%)		
T4	15 (39%)		
M1	5 (13%)		
			
*Non-BTC disease categories (*n*=33)*			
Benign biliary conditions^d^	18 (55%)		
Non-BTC malignancies^e^	8 (24%)		
PSC	7 (21%)		

BTC=biliary tract cancer; CA=carbohydrate antigen; CCA=cholangiocarcinoma; CEA=carcinoembryonic antigen; PSC=primary sclerosing cholangitis.

a Values are median with ranges.

b Student's *t*-test (two-tailed, unequal variances) or *χ*^2^ test applied.

c Assessment by means of imaging (pathological TNM stage available for one patient) (AJCC, 2002).

d Seven inflammatory and one ischaemic strictures, five sphincter-of-Oddi dysfunction and five choledocholithiasis.

e Four pancreatic cancer, one adenocarcinoma of unknown primary, one ovarian cancer, one B-cell lymphoma and one soft tissue spindle cell tumour.

**Table 2:** BTC prediction markers for biliary MUC4, serum MUC5AC and both mucin protein expressions combined on prospectively collected clinical specimens

	**Biliary MUC4 (*n*=62)**	**Serum MUC5AC (*n*=66)**	**Biliary MUC4 or serum MUC5AC (*n*=61)**
Sensitivity	27% (9/34)	44% (17/39)	58% (22/38)
Specificity	93% (26/28)	96% (26/27)	87% (20/23)
PPV	82% (9/11)	94% (17/18)	88% (22/25)
NPV	51% (26/51)	54% (26/48)	56% (20/36)

BTC=biliary tract cancer; NPV=negative predictive value; PPV=positive predictive value.
