# Supplementary material for: MUC4 and MUC5AC are highly specific tumour-associated mucins in biliary tract cancer
Source: Br J Cancer. 2008 May 13;98(10):1675–81. doi: 10.1038/sj.bjc.6604364 (PMC2391120; doi:10.1038/sj.bjc.6604364)
Supplement: Supplementary Figure [file 6604364x1.ppt]

## Slide 1
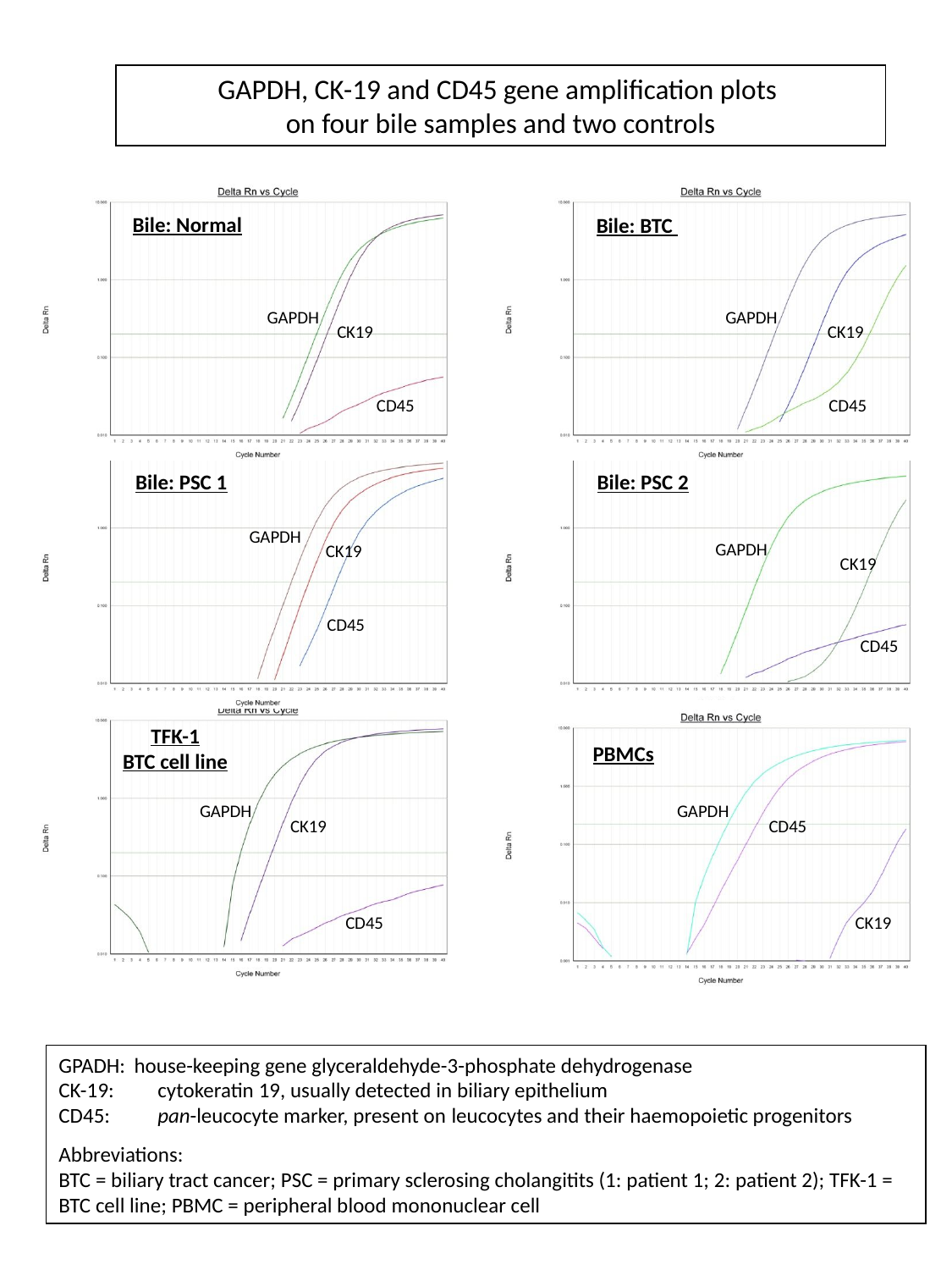

GAPDH, CK-19 and CD45 gene amplification plots
on four bile samples and two controls
Bile: Normal
Bile: BTC
GAPDH
GAPDH
CK19
CK19
CD45
CD45
Bile: PSC 1
Bile: PSC 2
GAPDH
GAPDH
CK19
CK19
CD45
CD45
TFK-1
BTC cell line
PBMCs
GAPDH
GAPDH
CK19
CD45
CD45
CK19
GPADH: 	house-keeping gene glyceraldehyde-3-phosphate dehydrogenase
CK-19: 		cytokeratin 19, usually detected in biliary epithelium
CD45: 		pan-leucocyte marker, present on leucocytes and their haemopoietic progenitors
Abbreviations:
BTC = biliary tract cancer; PSC = primary sclerosing cholangitits (1: patient 1; 2: patient 2); TFK-1 = BTC cell line; PBMC = peripheral blood mononuclear cell
